# Decline in *Cryptosporidium* Infection in Free-Ranging Rhesus Monkeys in a Park After Public Health Interventions

**DOI:** 10.3389/fcimb.2022.901766

**Published:** 2022-07-07

**Authors:** Ruilian Jia, Xi Wen, Yaqiong Guo, Lihua Xiao, Yaoyu Feng, Na Li

**Affiliations:** ^1^ State Key Laboratory of Bioreactor Engineering, School of Resources and Environmental Engineering, East China University of Science and Technology, Shanghai, China; ^2^ Center for Emerging and Zoonotic Diseases, College of Veterinary Medicine, South China Agricultural University, Guangzhou, China

**Keywords:** *Cryptosporidium* spp., subtype, nonhuman primates, rhesus monkey, intervention, surveillance

## Abstract

Nonhuman primates (NHPs) are considered an important source of parasitic zoonoses. A study in 2010 revealed high prevalence of *Cryptosporidium* spp. in free-ranging rhesus monkeys (*Macaca mulatta*) in a public park in Guiyang, southwestern China, which called for the control of disease in animals and long-term epidemiological tracking of *Cryptosporidium* spp. After the initiation of a series of public health interventions, we collected 2,402 fecal samples from monkeys and 123 water samples from lakes in the park on six occasions during 2013-2019. They were analyzed and genotyped for *Cryptosporidium* spp. using PCR and sequence analyses of the small subunit rRNA gene. The *C. hominis* and *C. parvum* identified were further subtyped by sequence analysis of the 60 kDa glycoprotein gene. Compared with the high prevalence of *Cryptosporidium* spp. in fecal samples (10.9% or 45/411) and water samples (47.8% or 11/23) in 2010, only 18 (0.7%) fecal samples and 3 (2.4%) water samples collected in the present study were positive for *Cryptosporidium* spp., including *C. hominis* (*n* = 9) and *C. parvum* (*n* = 12). The former belonged to the NHP-adapted IfA17G2R3 subtype, while the latter mostly belonged to rodent-adapted IIpA9. Therefore, the detection rate and genetic diversity of *Cryptosporidium* spp. during this study period were much lower than those before the public health interventions, and there was a switch from common occurrence of anthroponotic *C. hominis* subtypes to sporadic occurrence of NHP-adapted *C. hominis* and rodent-adapted *C. parvum* subtypes.

## Introduction

Cryptosporidiosis, an emerging infectious disease caused by *Cryptosporidium* spp., presents a major threat to global public health (Checkley et al., 2015). Up to date, over 40 *Cryptosporidium* species have been identified (Ryan et al., 2021b). Among them, approximately 20 have been detected in humans, but two species, *C. hominis* and *C. parvum*, are responsible for the majority of human cryptosporidiosis cases worldwide (Ryan et al., 2021a). As *Cryptosporidium* spp. are morphologically similar, it is essential to characterize the pathogens using molecular diagnostic tools for the identification of infection sources and transmission routes (Xiao and Feng, 2017).

Among the many reported reservoir animals, nonhuman primates (NHPs) have overlapped spectrum of *Cryptosporidium* species with humans due to their high genetic similarity as hosts (Li et al., 2011; Parsons et al., 2015; Devaux et al., 2019). As in humans, *C. hominis* and *C. parvum* are the dominant species found in NHPs. Various subtype families of these two major species have been detected, including mainly human-adapted subtype families (Ia, Ib, Id, Ie, and If of *C. hominis* and IIc of *C. parvum*) and NHP-adapted subtype families (Ii, Im, and In of *C. hominis* and IIo and IIp of *C. parvum*) (Feng and Xiao, 2017; Feng et al., 2018; Chen et al., 2019; Zhao et al., 2019). In addition, several other species such as *C. muris*, *C. andersoni*, *C. ubiquitum*, *C. meleagridis*, and *C. suis* as well as *C. parvum* zoonotic subtype families (IIa and IId), have also been detected in NHPs (Li et al., 2011; Karim et al., 2014; Sak et al., 2014; Du et al., 2015; Parsons et al., 2015; Chen et al., 2019; Zhao et al., 2019; Karim et al., 2021). Most of these species and subtypes have been reported in humans at varying frequencies.

Southwestern China has most NHPs in the country and Qianling Mountain Park in Guiyang is one of the popular urban-attractions for close interactions with free-ranging rhesus monkeys (*Macaca mulatta*). The park attracts thousands of visitors every day largely due to its urban location and large number of rhesus monkeys. Feeding monkeys is one of the main activities in the park. The abundant availability of food and uncontrolled growth had led to the significant increase in the monkey population since 2000. This was accompanied by the increased incidence of injuries of park visitors by monkeys. The park authority therefore initiated a series of intervention measures in controlling the population of monkeys in efforts to reduce human injuries and damages to the park environment by the animals.

We previously conducted a survey of *Cryptosporidium* spp. in rhesus monkeys at Qianling Mountain Park in November 2010 before the implementation of intervention measures (Ye et al., 2012). A high occurrence (10.9% or 45/411) of *Cryptosporidium* spp. was detected, with species and subtypes identified being commonly found in humans. Notably, the detection rates in different areas of the park correlated with the density of monkeys, and a high occurrence of the same *C. hominis* subtypes was detected in lake water in the park. As these subtypes are common ones in humans, it was suggested there was the likely bidirectional transmission of *Cryptosporidium* spp. between monkeys and humans.

In this study, we conducted a post-intervention monitoring of *Cryptosporidium* spp. in monkeys in Qianling Mountain Park and assessed the impact of interventions on the prevalence and genetic diversity of the pathogens in these animals.

## Materials and Methods

### The Park and Study Setting

Qianling Mountain Park, the public park covering an urban area of 4.26 square kilometers in Guiyang city, southwestern China, bring thousands of daily visitors for hiking and visiting the well-known rhesus monkeys. Over a thousand free-ranging monkeys live in the park and their main activity areas are around the Macaque Garden and Hongfu Temple. Visitors often offer food to monkeys from a short distance or play with them. In addition to monkeys and other wild animals, there is a zoo in the park. There are also several inter-connected small lakes in the park, where monkeys bathe in the summer.

### Intervention Measures and Animal Management in Qianling Mountain Park

Starting in 2011, in addition to the routine daily cleaning of the activity areas and removal of fecal materials, a series of intervention measures were implemented by the park staff and volunteers against two potential public health issues, the high density of monkeys and frequent contact between visitors and monkeys. They included: 1) increased public education on hazardous potential of feeding and contacting monkeys through broadcasting, posters and pamphlets; 2) special attentions to aggressive, injured, and sick monkeys including keeping them in quarantine when necessary; 3) controls of the monkey population size using various approaches; 4) expansion of activity space of monkeys by building climbing area among trees; 5) more stringent restrictions on irregular feeding of animals and provocation by park visitors; and 6) regular feeding of monkeys at fixed time and park locations.

### Sample Collection

From August 2013 to October 2019, a total of 2,402 fecal samples were collected from rhesus monkeys and 123 water samples from park lakes in Qianling Mountain Park on six occasions (**Table 1**). The fecal samples consisted of fresh dropping from monkeys while water samples were turbid water collected from different sites (one 500-mL grab samples per site) of the lakes within the park. The sampling size of fecal samples was strictly controlled not to exceed one third of the estimated total number of monkeys at each sampling site. The fecal and water samples were placed into individual clean containers and transported to the laboratory in coolers with ice packs. Water samples were concentrated by centrifugation at 2000 × *g* for 20 min. The concentrate and fecal samples were stored at 4°C in 2.5% potassium dichromate solution before DNA extraction.

**Table 1:** Detection rates and distribution of *Cryptosporidium* species and subtypes in samples from rhesus monkeys and water in Qianling Mountain Park, Guiyang, China.

Sampling time	Sample type	Sample size	No. positive (%)	*Cryptosporidium* spp.	*Cryptosporidium* subtype	Reference
November 2010	Feces	411	45 (10.9)	*C. hominis* (39), *C. parvum* (5), *C. felis* (1)	IaA13R8 (8), IaA13R7 (2), IaA14R7 (2), IdA20 (13), IeA11G3T3 (13), IfA17G2R5 (1), IIcA5G3a (5)	(Ye et al., 2012)
	Water	23	11 (47.8)	*C. hominis* (11)	IaA13R8 (2), IeA11G3T3 (7)	
March 2013	Feces	374	6 (1.6)	*C. parvum* (6)	IIpA9 (3)	This study
	Water	55	3 (5.5)	*C. parvum* (3)	IIpA9 (2)	
August 2013	Feces	162	0 (0)	-	-	
April 2018	Feces	389	3 (0.8)	*C. parvum* (3)	IIdA15G1 (1)	
March 2019	Feces	478	8 (1.7)	*C. hominis* (8)	IfA17G2R3 (5)	
July 2019	Feces	488	1 (0.2)	*C. hominis* (1)	IfA17G2R3 (1)	
	Water	34	0 (0)	-	-	
October 2019	Feces	511	0 (0)	-	-	
	Water	34	0 (0)	-	-	
Total	Feces	2402	18 (0.7)	*C. hominis* (9), *C. parvum* (9)	IfA17G2R3 (6), IIpA9 (3), IIdA15G1 (1)	
	Water	123	3 (2.4)	*C. parvum* (3)	IIpA9 (2)	

### DNA Extraction

Prior to DNA extraction, fecal samples and water concentrate were washed three times with distilled water by centrifugation at 2000 × *g* for 10 min to remove potassium dichromate. Genomic DNA was extracted from them using FastDNA Spin Kit for Soil (MP Biomedical, Santa Ana, CA, USA) and stored at − 20°C.

### *Cryptosporidium* Detection, Genotyping and Subtyping

Detection of *Cryptosporidium* spp. was made from extracted genomic DNA using nested PCR amplification of a ~830 bp fragment of the small subunit (SSU) rRNA gene (Xiao et al., 1999). Positive PCR products were sequenced to identify the species. The subtype identification of *Cryptosporidium* spp. were conducted by nested PCR amplification of a ~850 bp fragment of the 60 kDa glycoprotein (*gp60*) gene and sequence analysis of the PCR product (Feng et al., 2009). Genomic DNA of *C. bovis* and *C. tyzzeri* was used as the positive control for the SSU rRNA PCR and the *gp60* PCR, respectively, while reagent-grade water was used as the negative control.

### Sequence Analysis and Phylogenetic Analysis

All positive secondary PCR products were sequenced in both directions on an ABI 3730 Genetic Analyzer (Applied Biosystems, Foster City, CA, USA). The nucleotide sequences obtained were assembled using ChromasPro 2.1.8.0 (http://technelysium.com.au/ChromasPro.html), edited using BioEdit 7.1.3.0 (http://www.mbio.ncsu.edu/BioEdit/bioedit.html), and aligned with reference sequences from GenBank using ClustalX 2.0.12 (http://clustal.org). A maximum likelihood (ML) tree based on the *gp60* sequences (688 - 1014 bp) was constructed to assess the phylogenetic relationships among subtypes of *C. hominis* and *C. parvum* from this study and reference sequences from GenBank using MEGA X (https://www.megasoftware.net/) based on substitution rates calculated with GTR+Γ+I mode (Gamma = 2.29) and bootstrapping with 1,000 replicates.

## Results

### Occurrence of *Cryptosporidium spp*. in Rhesus Monkeys

Of the 2,402 fecal samples from rhesus monkeys and 123 water samples from lakes in Qianling Mountain Park during 2013 to 2019, 18 fecal samples (0.7%) and 3 water samples (2.4%) were positive for *Cryptosporidium* spp.

The detection rates of *Cryptosporidium* spp. in fecal samples were 1.6% (6/374), 0.0% (0/162), 0.8% (3/389), 1.7% (8/478), 0.2% (1/488), and 0.0% (0/511) in March 2013, August 2013, April 2018, March 2019, July 2019 and October 2019, respectively (**Table 1**). The overall detection rate of *Cryptosporidium* spp. in fecal samples collected in this study (0.7% or 18/2402) was much lower than what was examined in 2010 (10.9% or 45/411).

Only water samples collected in March 2013 were positive for *Cryptosporidium* spp. with a 5.5% detection rate (3/55). In contrast, water samples collected in July 2019 (0/34) and October 2019 (0/34) were negative (**Table 1**). The overall detection rate of *Cryptosporidium* spp. in water samples collected in this study (2.4%, 3/123) was also much lower than in 2010 (47.8%, 11/23).

### *Cryptosporidium* Species

Among the 21 *Cryptosporidium*-positive samples (18 fecal samples and 3 water samples), sequence analysis of the SSU rRNA PCR products identified *C. hominis* (*n* = 9) and *C. parvum* (*n* = 12). *Cryptosporidium hominis* was detected in nine fecal samples, while *C. parvum* was detected in nine fecal samples and three water samples.

**Figure 1:**
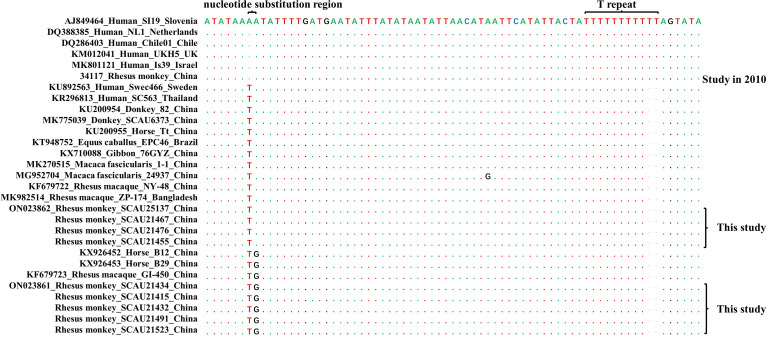
Sequences of hypervariable region of the small subunit rRNA gene among *Cryptosporidium hominis* isolates from various hosts. Compared with the reference sequence AJ849464 (Soba et al., 2006), identical nucleotides and nucleotide deletions are present as dots and cashes, respectively. The two regions with nucleotide substitutions and differences in the number of consecutive T are labeled. Sequences from this and previous studies are indicated. In this study, all samples used in the analysis are from monkey feces.

Two types of nucleotide sequences of the SSU rRNA gene were obtained from *C. hominis* in this study. One type detected in four monkeys was identical to KF679722, KU200954 and MK982514 obtained from various NHPs and equine animals in China and other Asian countries (Karim et al., 2014; Karim et al., 2021). The other type detected in five monkeys was identical to KF679723, KX926452 and KX926453 obtained from rhesus macaques and horses in China (Karim et al., 2014; Deng et al., 2017). The two types of sequences differed from each other by one nucleotide (A or T) in the hypervariable region of the SSU rRNA gene. A close inspection of the trace files indicated the presence of both nucleotide at the position. These two types of sequences were slightly different from the common SSU rRNA sequences (AJ849464 and many others) obtained from *C. hominis* in humans worldwide and rhesus monkeys in the study park examined in 2010. They had an A-to-T nucleotide substitution in all sequences, an A-to-G nucleotide substitution in some sequences, and nine consecutive T instead of 11 in the hypervariable region downstream from the substitutions (**Figure 1**).

The nucleotide sequences of the SSU rRNA gene from *C. parvum* were identical to each other and to the reference sequences KC885894, MK731961, MK956932 and MW092529 obtained from bamboo rats in various areas in China (Liu et al., 2015; Wei et al., 2019; Li et al., 2020a; Li et al., 2020b). It had an A-to-T nucleotide substitution in the hypervariable region from sequences from the common *C. parvum* in humans, cattle and other animals as described previously (Li et al., 2020a).

### *Cryptosporidium* Hominis and *C. Parvum* Subtypes

Among the 21 *Cryptosporidium*-positive samples, 12 of them were successfully amplified and sequenced at the *gp60* locus.

Only one subtype was seen in the six fecal samples of *C. hominis* subtyped, namely IfA17G2R3. The nucleotide sequences obtained were identical to each other and has only one nucleotide difference from a sequence (JX000570) obtained from monkeys in the same park in the non-repeat nucleotide region (Ye et al., 2012). As all sequences of the If subtype family in GenBank has G instead of A in JX000570, this sequence difference could be due to a sequencing error in JX000570.

Two subtypes were seen in the six *C. parvum* samples subtyped successfully, including IIdA15G1 (in one fecal sample collected in April 2018) and IIpA9 (in three fecal and two water samples collected in March 2013). The sequences of IIpA9 were identical to KC885904 and other *gp60* sequences obtained from bamboo rats in China (Liu et al., 2015; Wei et al., 2019; Li et al., 2020a).

**Figure 2:**
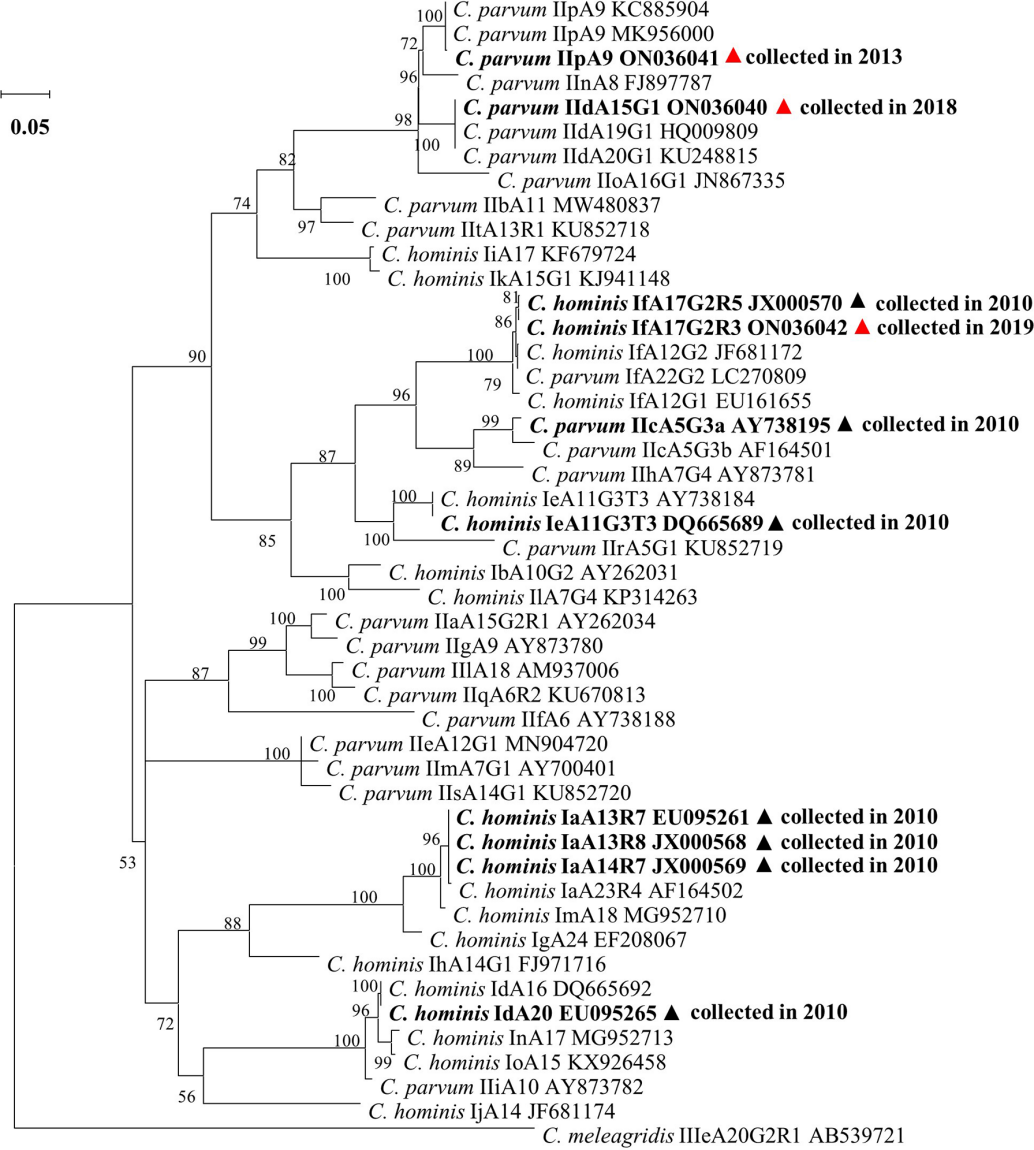
Phylogenetic relationship of *Cryptosporidium hominis* and *Cryptosporidium parvum* subtypes based on a maximum likelihood (ML) analysis of sequences of the 60 kDa glycoprotein gene from collected samples and compared to the GenBank reference gene, with bootstrap values above 50% being shown on nodes. Isolates from this study are in bold and labeled with red triangles, while isolates from the study conducted in 2010 are in bold and labeled with black triangles. In this study, all samples used in the analysis are from monkey feces.

In phylogenetic analysis of the *gp60* sequences obtained from the study, IfA17G2R3 clustered with other If subtypes while IIpA9 clustered with IIdA15G1 and other IId and IIn subtypes (**Figure 2**).

## Discussion

In this study, we have conducted a post-intervention surveillance on the prevalence of *Cryptosporidium* spp. in rhesus monkeys in Qianling Mountain Park, southwest China. The occurrence and genetic diversity of *Cryptosporidium* spp. have declined compared with those in a study conducted prior to the intervention in 2010. In addition, the *C. hominis* and *C. parvum* subtypes have changed from anthroponotic ones to NHP-adapted *C. hominis* and rodent-adapted *C. parvum* subtypes.

**Figure 3:**
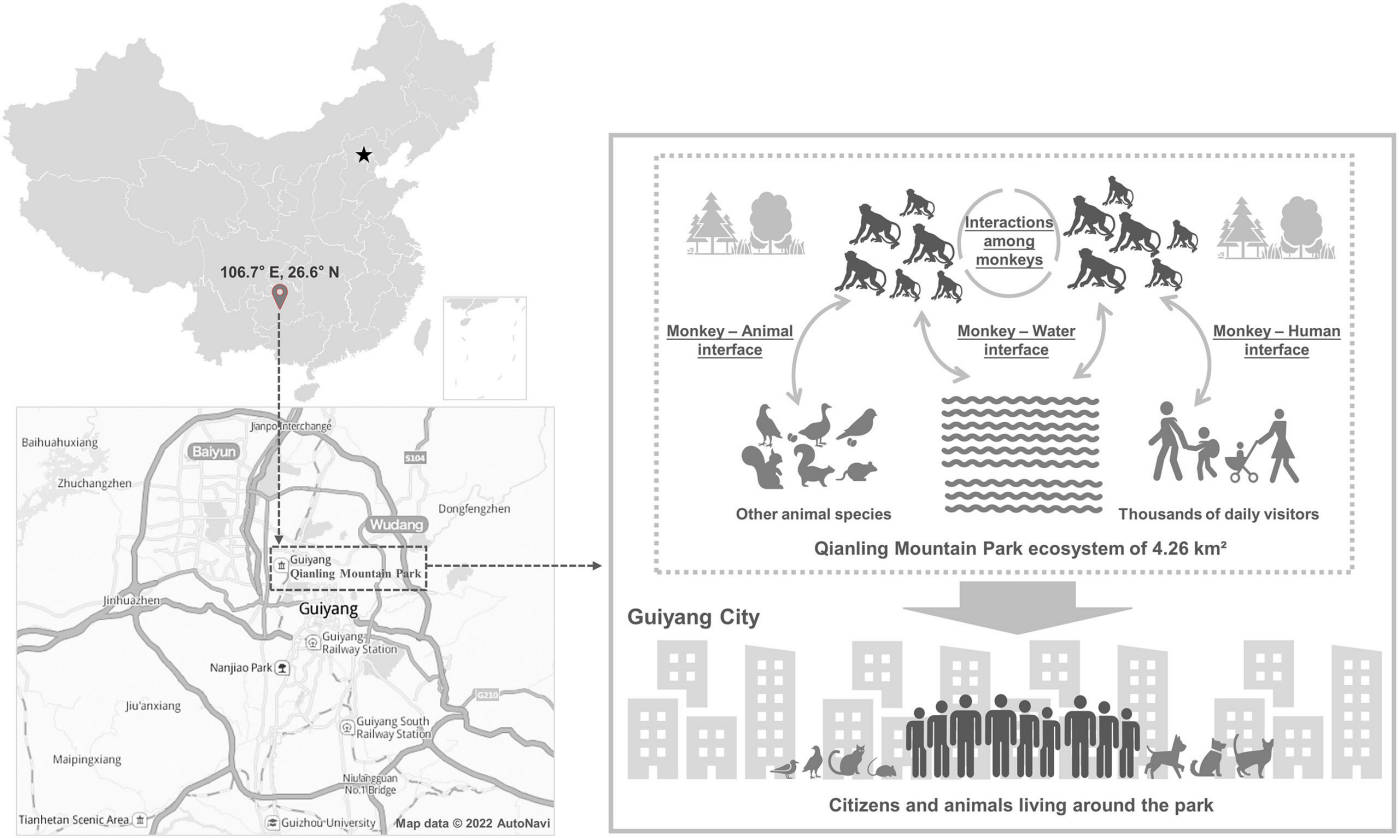
Schematic overview of the location and ecosystem of Qianling Mountain Park, main One Health interfaces, and potential public health implications. The star symbol represents Beijing city, the capital of China.

Intervention measures implemented in recent years could have played a role in reducing the *Cryptosporidium* detection rate of rhesus monkeys in Qianling Mountain Park. As shown in **Figure 3**, the mild subtropical climate, connected urban surface water system, large number of monkeys and visitors, and frequent interactions between humans and monkeys might facilitate *Cryptosporidium* transmission in this park (Hassell et al., 2017; Devaux et al., 2019; Pozio, 2020). Nevertheless, compared with the 10.9% detection rate of *Cryptosporidium* spp. in fecal samples from rhesus monkeys in 2010, only 0.7% samples collected during 2013-2019 were positive for *Cryptosporidium* spp. Animal density itself might not have any effect on reducing the occurrence of *Cryptosporidium* spp. in these animals, as the number of animals in the park roughly doubled since 2010 due to resistance from the residents and park visitors to many of the animal control activities implemented by the park management. It is possible that the increased sanitation, reduced contact between monkeys and humans as part of the prevention of injuries of park visitors, and quarantine of sick and young animals might have contributed to the decrease in occurrence of cryptosporidiosis. As enteric pathogens transmitted through the fecal-oral route, these practices are recommended as major control measures against *Cryptosporidiu*m spp. in humans (Lin et al., 2018; Ross et al., 2020). Sustained interventions are needed to prevent the rebound of *Cryptosporidium* infections in rhesus monkeys in the park.

The shift in dominant *Cryptosporidium* species/subtypes in rhesus monkeys might be related to the effectiveness of the intervention measures in controlling the transmission of zoonotic pathogens. In the previous study conducted in 2010, the *C. hominis* identified had SSU rRNA sequences identical to those from isolates from humans, with dominant subtype families belonging to Ia (12/39), Id (13/39), and Ie (13/39). In agreement with this, the *C. parvum* identified also belonged to the anthroponotic IIc subtype family (Ye et al., 2012). In contrast, the *C. hominis*- and *C. parvum*- positive samples in this study generated SSU rRNA sequences identical to those normally seen in host-adapted subtype families found in NHPs and rodents, respectively. Although the *C. hominis* subtype IfA17G2R3 detected in a few monkeys in 2019 was seen in one monkey in 2010, it had generated SSU rRNA sequences identical to those seen in subtypes in NHPs (Feng et al., 2018; Chen et al., 2019). The IIp subtype of *C. parvum* identified in a few monkeys in the present study also has unique SSU rRNA sequences and represents a well-known host-adapted pathogen in bamboo rats in China (Liu et al., 2015; Feng et al., 2018; Wei et al., 2019; Li et al., 2020a; Li et al., 2020b). The nucleotide differences in the SSU rRNA sequences of *C. hominis* and *C. parvum* in rhesus monkeys over the time in the park, might be due to genetic recombination driven by host-adaptive evolution (Feng et al., 2018). During this long-term monitoring, the *C. hominis* and *C. parvum* subtypes have shifted from anthroponotic ones to zoonotic ones. The latter might have been obtained from other free-ranging wildlife living in same park ecosystem.

Despite their occasional occurrence, attention should be directed to the potential emergence of these unusual *C. hominis* and *C. parvum* subtypes in humans. For instance, the If subtypes of *C. hominis* is commonly found in humans worldwide (Yang et al., 2021). Several other NHP-adapted *C. hominis* subtypes have been found in humans in several countries (Lebbad et al., 2021). Another rodent-adapted *C. parvum* subtype family generated related to IIp, IIo, has also been found in humans in Southeast Asia and New Zealand (Sannella et al., 2019; Garcia et al., 2020). These divergent *C. hominis* and *C. parvum* subtypes are increasingly detected in animals in China (Guo et al., 2021), indicating the possible emergence of them in humans in near future. Thus, further studies involving expanded sampling of park visitors and residents surrounding this park are needed to elucidate the role of free-ranging rhesus monkeys in the epidemiology of human cryptosporidiosis.

From One Health perspective, real-time surveillance of the pathogens in rhesus monkeys in Qianling Mountain Park and larger-scale epidemiological investigations in other free-ranging wildlife living in the same park ecosystem are necessary to track the sources and routes of *Cryptosporidium* transmission in this natural habitat for early warning and intervention. Further studies on genetic structure and biological characteristics of the unique *C. hominis* and *C. parvum* subtypes present in animals, as well as in humans from surrounding communities are needed to understand their impact on public health. Additionally, a rigorous longitudinal study can assess effectiveness of occurrence of intervention measures on *Cryptosporidium* occurrence in free-range rhesus monkeys in Qianling Mountain Park.

## Data Availability Statement

Representative nucleotide sequences in this study were deposited in GenBank under accession numbers ON023860-ON023862 and ON036040-ON036042.

## Ethics Statement

The study protocol was approved by the Research Ethics Committee of the East China University of Science and Technology and South China Agricultural University. Consent for sample collections was obtained from the park management. The research was conducted in compliance with the Chinese Laboratory Animal Administration Act of 2017. The sampling did not involve any handling of the animals.

## Author Contributions

YF and NL conceived and designed the experiments. RJ and XW performed the experiments and analyzed the data. YG and LX provided technical assistance. RJ, LX, YF and NL wrote the manuscript. All authors contributed to the article and approved the submitted version.

## Funding

This work was supported in part by the National Natural Science Foundation of China (U1901208 and 32030109), Guangdong Major Project of Basic and Applied Basic Research (2020B0301030007), 111 Project (D20008), and Innovation Team Project of Guangdong University (2019KCXTD001).

## Conflict of Interest

The authors declare that the research was conducted in the absence of any commercial or financial relationships that could be construed as a potential conflict of interest.

## Publisher’s Note

All claims expressed in this article are solely those of the authors and do not necessarily represent those of their affiliated organizations, or those of the publisher, the editors and the reviewers. Any product that may be evaluated in this article, or claim that may be made by its manufacturer, is not guaranteed or endorsed by the publisher.
